# Supplementary material for: Gut Microbiota as Emerging Players in the Development of Alcohol-Related Liver Disease
Source: Biomedicines. 2024 Dec 31;13(1):74. doi: 10.3390/biomedicines13010074 (PMC11761646; doi:10.3390/biomedicines13010074)
Supplement: Supplementary file 1 [file biomedicines-13-00074-s001.zip › biomedicines-3387047-supplementary.pdf]

We temporarily define Table S1 as a supplementary table for the section of treatment.

Table S1. Overview of the potential therapeutic approaches in ALD.

Intervention/therapy	Subjects	Dose and method	Result and conclusion	Study Design
Probiotics				
Bifidobacterium breve ATCC15700 (Tian X et al. 2020)[180]	Mice exposed to chronic alcohol.	5 × 10^{10}CFU; For 6 weeks; Oral gavage	Prevented liver steatosis and inflammation, maintained immune homeostasis, improved intestinal barrier function, and modulated gut microbiota.	Preclinical study
VSL#3(*Lactobacillus*, *Bifidobacterium*, and *Streptococcus thermophilus*) (Chang B et al.2013)[182]	Mice with acute alcohol liver disease	0.6 g/kg body weight; Intra-gastric gavage	Regulated the ecological balance of the gut microflora, prevented passage of endotoxin and other bacterial products from the gut lumen into the portal circulation and down-regulated the expression of TNFα.	Preclinical study
Lactobacillus rhamnosus Gorbach-Goldin (LGG) （Forsyth C B et al.2009)[183]	Mice model of ALD.	2.5×10^7 live Lactobacillus GG; For 10 weeks; Oral gavage.	Reduced the severity of alcoholic steatohepatitis, alcohol-induced gut hyperpermeability, and alcohol-induced tissue (gut and liver) and systemic oxidative stress.	Preclinical study
Lactobacillus rhamnosus GG (LGG) (Zhu Y et al.2022)[184]	Mice model of alcohol-induced liver injury	1×10^9 CFU; For 4 weeks; Oral gavage.	Ameliorated liver enzymes, steatosis and inflammation; Restored IFN-γ-producing CD4 T (Th1) cells	Preclinical study
Lactobacillus rhamnosus GG (Wang Y et al.2013)[185]	Mice with alcoholic liver inflammation	1×10^9 CFU; For 2 weeks; Oral gavage	Reduced hepatic inflammation and liver injury and markedly reduced TNFα expression.	Preclinical study
Lactobacillus plantarum (TSP05), *Lactobacillus fermentum* (TSF331) and *Lactobacillus reuteri* (TSR332) (Ps H et al.2021)[186]	Mice model of alcoholic steatohepatitis	8.2x10^9 CFU; For 4 weeks; Oral gavage	Neutralized free radicals and displayed high antioxidant activity in vitro; demonstrated significantly lower ALT, AST and triglyceride levels; Downregulated the proinflammatory cytokines TNF-α and IL-6; Upregulated glutathione and glutathione peroxidase activity.	Preclinical study
Lactobacillus rhamnosus R0011 and *Lactobacillus acidophilus* R0052 (Bang C S et al.2014)[187]	Mice models of ALD	1 mg/mL; For 4 weeks; Oral gavage.	Decreased interleukin-1β levels in liver tissues	Preclinical study
Lactobacillus rhamnosus R0011 and *acidophilus* R0052	Mice models of ALD	1 mg/mL; For 2 weeks;	Down-regulated TLR 4 expression.	Preclinical study

Intervention/therapy	Subjects	Dose and method	Result and conclusion	Study Design
(Hong M et al.2015)[188]		Intra-gastric gavage.		
Lactobacillus rhamnosus NKU FL1-8 (Liu H et al.2024)[189]	Mice models of ALD	5×10^9 CFU; For three weeks; Oral gavage.	Increased the relative abundance of *Lactobacillus*, *Ruminococcaceae*; Reduced LPS and enhanced intestinal tight junction proteins	Preclinical study
Lactobacillus reuteri （Cheng Y et al. 2022)[191]	Mice models of ALD	2×10^7 CFU; For 18 days; Oral gavage	Alleviated the lipid accumulation of ALD	Preclinical study
Pediococcus pentosaceus (Jiang X W et al.2020)[165]	Mice models of ALD	2×10^9 CFUs; Only once; Oral gavage.	Improved ethanol-induced liver injury, with lower ALT, AST and TG levels; Decreased neutrophil infiltration.	Preclinical study
Phocaeicola dorei and *Lactobacillus helveticus* (Eom J A et al.2023)[13]	Mice chronic-plus-binge alcohol feeding model	10^9 CFU For a week; Oral gavage.	Improved liver inflammation and intestinal barrier damage; Increased NK cell activity.	Preclinical study
Akkermansia muciniphila (Grander C et al. 2018)[47]	Mice, acute model of ethanol toxicity	1.5×10^9 CFU For 3 days; Oral gavage.	Ameliorated hepatic injury and neutrophil infiltration	Preclinical study
Phage				
Phage cocktail (*E. faecalis*) (Duan Y et al.2019)[43]	Humanized mice colonized with fecal microbiota from alcoholic hepatitis patients.	10^{10} PFU Oral administration	Reduced cytolysin levels in the liver; Prevented ethanol-induced liver damage in humanized mice.	Preclinical study

CFU, colony-forming unit; PFU, plaque-forming unit; *E. faecalis*, *Enterococcus faecalis*; LPS, lipopolysaccharides; ALT, alanine aminotransferase; AST, aspartate aminotransferase; ALD, alcoholic-related liver disease; AH, alcoholic hepatitis; LBP, lipopolysaccharide-binding protein; GGT, gamma-glutamyl transferase; TG, triglycerides;.

References

[11] Kirpich I A, Solovieva N V, Leikhter S N, Shidakova N A, Lebedeva O V, Sidorov P I, Bazhukova T A, Soloviev A G, Barve S S, McClain C J, Cave M. Probiotics restore bowel flora and improve liver enzymes in human alcohol-induced liver injury: a pilot study[J/OL]. Alcohol (Fayetteville, N.Y.), 2008, 42(8): 675-682. DOI:10.1016/j.alcohol.2008.08.006.

[13] Eom J A, Jeong J J, Han S H, Kwon G H, Lee K J, Gupta H, Sharma S P, Won S M, Oh K K, Yoon S J, Joung H C, Kim K H, Kim D J, Suk K T. Gut-microbiota prompt activation of natural killer cell on alcoholic liver disease[J/OL]. Gut Microbes, 2023, 15(2): 2281014. DOI:10.1080/19490976.2023.2281014.

[47] Grander C, Adolph T E, Wieser V, Lowe P, Wrzosek L, Gyongyosi B, Ward D V, Grabherr F, Gerner R R, Pfister A, Enrich B, Ciocan D, Macheiner S, Mayr L, Drach M, Moser P, Moschen A R, Perlemuter G, Szabo G, Cassard A M, Tilg H. Recovery of ethanol-induced Akkermansia muciniphila depletion ameliorates alcoholic liver disease[J/OL]. Gut, 2018, 67(5): 891-901. DOI:10.1136/gutjnl-2016-313432.

[43] Duan Y, Llorente C, Lang S, Brandl K, Chu H, Jiang L, White R C, Clarke T H, Nguyen K, Torralba M, Shao Y, Liu J, Her-nandez-Morales A, Lessor L, Rahman I R, Miyamoto Y, Ly M, Gao B, Sun W, Kiesel R, Hutmacher F, Lee S, Ventura-Cots M, Bosques-Padilla F, Verna E C, Abraldes J G, Brown R S, Vargas V, Altamirano J, Caballería J, Shawcross D L, Ho S B, Louvet A, Lucey M R, Mathurin P, Garcia-Tsao G, Bataller R, Tu X M, Eckmann L, van der Donk W A, Young R, Lawley T D, Stärkel P, Pride D, Fouts D E, Schnabl B. Bacteriophage targeting of gut bacterium attenuates alcoholic liver disease[J/OL]. Nature, 2019, 575(7783): 505-511. DOI:10.1038/s41586-019-1742-x.

[159] Horvath A, Durdevic M, Leber B, di Vora K, Rainer F, Krones E, Douschan P, Spindelboeck W, Durchschein F, Zollner G, Stauber R E, Fickert P, Stiegler P, Stadlbauer V. Changes in the Intestinal Microbiome during a Multispecies Probiotic Intervention in Compensated Cirrhosis[J/OL]. Nutrients, 2020, 12(6): 1874. DOI:10.3390/nu12061874.

[160] Li X, Liu Y, Guo X, Ma Y, Zhang H, Liang H. Effect of Lactobacillus casei on lipid metabolism and intestinal microflora in patients with alcoholic liver injury[J/OL]. European Journal of Clinical Nutrition, 2021, 75(8): 1227-1236. DOI:10.1038/s41430-020-00852-8.

[161] Stadlbauer V, Mookerjee R P, Hodges S, Wright G A K, Davies N A, Jalan R. Effect of probiotic treatment on deranged neutrophil function and cytokine responses in patients with compensated alcoholic cirrhosis[J/OL]. Journal of Hepatology, 2008, 48(6): 945-951. DOI:10.1016/j.jhep.2008.02.015.

[162] J M, F F, E G L, H L, H J, R S, K S, S F, J M, A M, Ij C, L T, N D, R W, R M, G W, R J. A Double-Blind, Randomized Place-bo-Controlled Trial of Probiotic Lactobacillus casei Shirota in Stable Cirrhotic Patients[J/OL]. Nutrients, 2020, 12(6). DOI:10.3390/nu12061651.

[163] Lata J, Novotný I, Príbramská V, Juránková J, Fric P, Kroupa R, Stibůrek O. The effect of probiotics on gut flora, level of en-dotoxin and Child-Pugh score in cirrhotic patients: results of a double-blind randomized study[J/OL]. European Journal of Gastroenterology & Hepatology, 2007, 19(12): 1111-1113. DOI:10.1097/MEG.0b013e3282efa40e.

[164] Han S H, Suk K T, Kim D J, Kim M Y, Baik S K, Kim Y D, Cheon G J, Choi D H, Ham Y L, Shin D H, Kim E J. Effects of probiotics (cultured Lactobacillus subtilis/Streptococcus faecium) in the treatment of alcoholic hepatitis: randomized-controlled multi-center study[J/OL]. European Journal of Gastroenterology & Hepatology, 2015, 27(11): 1300-1306. DOI:10.1097/MEG.0000000000000458.

[165] Bajaj J S, Gavis E A, Fagan A, Wade J B, Thacker L R, Fuchs M, Patel S, Davis B, Meador J, Puri P, Sikaroodi M, Gillevet P M. A Randomized Clinical Trial of Fecal Microbiota Transplant for Alcohol Use Disorder[J/OL]. Hepatology (Baltimore, Md.), 2021, 73(5): 1688-1700. DOI:10.1002/hep.31496.

[166] Philips C A, Pande A, Shasthry S M, Jamwal K D, Khillan V, Chandel S S, Kumar G, Sharma M K, Maiwall R, Jindal A, Choudhary A, Hussain M S, Sharma S, Sarin S K. Healthy Donor Fecal Microbiota Transplantation in Steroid-Ineligible Severe Alcoholic Hepatitis: A Pilot Study[J/OL]. Clinical Gastroenterology and Hepatology, 2017, 15(4): 600-602. DOI:10.1016/j.cgh.2016.10.029.

[167] Bajaj J S, Kakiyama G, Savidge T, Takei H, Kassam Z A, Fagan A, Gavis E A, Pandak W M, Nittono H, Hylemon P B, Boonma P, Haag A, Heuman D M, Fuchs M, John B, Sikaroodi M, Gillevet P M. Antibiotic-Associated Disruption of Microbiota Composi-tion and Function in Cirrhosis Is Restored by Fecal Transplant[J/OL]. Hepatology (Baltimore, Md.), 2018, 68(4): 1549-1558. DOI:10.1002/hep.30037.

[168] Bajaj J S, Salzman N H, Acharya C, Sterling R K, White M B, Gavis E A, Fagan A, Hayward M, Holtz M L, Matherly S, Lee H, Osman M, Siddiqui M S, Fuchs M, Puri P, Sikaroodi M, Gillevet P M. Fecal Microbial Transplant Capsules Are Safe in Hepatic Encephalopathy: A Phase 1, Randomized, Placebo-Controlled Trial[J/OL]. Hepatology (Baltimore, Md.), 2019, 70(5): 1690-1703. DOI:10.1002/hep.30690.

[169] Sharma A, Roy A, Premkumar M, Verma N, Duseja A, Taneja S, Grover S, Chopra M, Dhiman R K. Fecal microbiota trans-plantation in alcohol-associated acute-on-chronic liver failure: an open-label clinical trial[J/OL]. Hepatology International, 2022, 16(2): 433-446. DOI:10.1007/s12072-022-10312-z.

[179] Tian X, Li R, Jiang Y, Zhao F, Yu Z, Wang Y, Dong Z, Liu P, Li X. Bifidobacterium breve ATCC15700 pretreatment prevents alcoholic liver disease through modulating gut microbiota in mice exposed to chronic alcohol intake[J/OL]. Journal of Functional Foods, 2020, 72: 104045. DOI:10.1016/j.jff.2020.104045.

[180] Chang B, Sang L, Wang Y, Tong J, Zhang D, Wang B. The protective effect of VSL#3 on intestinal permeability in a rat model of alcoholic intestinal injury[J/OL]. BMC gastroenterology, 2013, 13: 151. DOI:10.1186/1471-230X-13-151.

[181] Forsyth C B, Farhadi A, Jakate S M, Tang Y, Shaikh M, Keshavarzian A. Lactobacillus GG treatment ameliorates alco-hol-induced intestinal oxidative stress, gut leakiness, and liver injury in a rat model of alcoholic steatohepatitis[J/OL]. Alcohol, 2009, 43(2): 163-172. DOI:10.1016/j.alcohol.2008.12.009.

[182] Zhu Y, Wang X, Zhu L, Tu Y, Chen W, Gong L, Pan T, Lin H, Lin J, Sun H, Ge Y, Wei L, Guo Y, Lu C, Chen Y, Xu L. Lactobacillus rhamnosus GG combined with inosine ameliorates alcohol-induced liver injury through regulation of intestinal barrier and Treg/Th1 cells[J/OL]. Toxicology and Applied Pharmacology, 2022, 439: 115923. DOI:10.1016/j.taap.2022.115923.

[183] Wang Y, Liu Y, Kirpich I, Ma Z, Wang C, Zhang M, Suttles J, McClain C, Feng W. Lactobacillus rhamnosus GG reduces hepatic TNFα production and inflammation in chronic alcohol-induced liver injury[J/OL]. The Journal of Nutritional Biochemistry, 2013, 24(9): 1609-1615. DOI:10.1016/j.jnutbio.2013.02.001.

[184] Ps H, Cw C, Yw K, Hh H. Lactobacillus spp. reduces ethanol-induced liver oxidative stress and inflammation in a mouse model of alcoholic steatohepatitis[J/OL]. Experimental and therapeutic medicine, 2021, 21(3). DOI:10.3892/etm.2021.9619.

[185] Bang C S, Hong S H, Suk K T, Kim J B, Han S H, Sung H, Kim E J, Kim M J, Kim M Y, Baik S K, Kim D J. Effects of Korean Red Ginseng (Panax ginseng), urushiol (Rhus vernicifera Stokes), and probiotics (Lactobacillus rhamnosus R0011 and Lactobacillus acidophilus R0052) on the gut-liver axis of alcoholic liver disease[J/OL]. Journal of Ginseng Research, 2014, 38(3): 167-172. DOI:10.1016/j.jgr.2014.04.002.

[186] Hong M, Kim S W, Han S H, Kim D J, Suk K T, Kim Y S, Kim M J, Kim M Y, Baik S K, Ham Y L. Probiotics (Lactobacillus rhamnosus R0011 and acidophilus R0052) reduce the expression of toll-like receptor 4 in mice with alcoholic liver disease[J/OL]. PloS One, 2015, 10(2): e0117451. DOI:10.1371/journal.pone.0117451.

[187] Liu H, Fan D, Wang J, Wang Y, Li A, Wu S, Zhang B, Liu J, Wang S. Lactobacillus rhamnosus NKU FL1-8 Isolated from Infant Feces Ameliorates the Alcoholic Liver Damage by Regulating the Gut Microbiota and Intestinal Barrier in C57BL/6J Mice[J/OL]. Nutrients, 2024, 16(13): 2139. DOI:10.3390/nu16132139.

[189] Cheng Y, Xiang X, Liu C, Cai T, Li T, Chen Y, Bai J, Shi H, Zheng T, Huang M, Fu W. Transcriptomic Analysis Reveals Lactobacillus reuteri Alleviating Alcohol-Induced Liver Injury in Mice by Enhancing the Farnesoid X Receptor Signaling Pathway[J/OL]. Journal of Agricultural and Food Chemistry, 2022, 70(39): 12550-12564. DOI:10.1021/acs.jafc.2c05591.

[190] Jiang X W, Li Y T, Ye J Z, Lv L X, Yang L Y, Bian X Y, Wu W R, Wu J J, Shi D, Wang Q, Fang D Q, Wang K C, Wang Q Q, Lu Y M, Xie J J, Li L J. New strain of Pediococcus pentosaceus alleviates ethanol-induced liver injury by modulating the gut microbiota and short-chain fatty acid metabolism[J/OL]. World Journal of Gastroenterology, 2020, 26(40): 6224-6240. DOI:10.3748/wjg.v26.i40.6224.

[191] Philips C A, Phadke N, Ganesan K, Ranade S, Augustine P. Corticosteroids, nutrition, pentoxifylline, or fecal microbiota transplantation for severe alcoholic hepatitis[J/OL]. Indian Journal of Gastroenterology: Official Journal of the Indian Society of Gastroenterology, 2018, 37(3): 215-225. DOI:10.1007/s12664-018-0859-4.

[192] Abedon S T, García P, Mullany P, Aminov R. Editorial: Phage Therapy: Past, Present and Future[J/OL]. Frontiers in Microbiology, 2017, 8: 981. DOI:10.3389/fmicb.2017.00981.
